# Proton magnetic resonance spectroscopy in skeletal muscle: Experts' consensus recommendations

**DOI:** 10.1002/nbm.4266

**Published:** 2020-02-05

**Authors:** Martin Krššák, Lucas Lindeboom, Vera Schrauwen‐Hinderling, Lidia S. Szczepaniak, Wim Derave, Jesper Lundbom, Douglas Befroy, Fritz Schick, Jürgen Machann, Roland Kreis, Chris Boesch

**Affiliations:** ^1^ Division of Endocrinology and Metabolism, Department of Internal Medicine III & High Field MR Centre, Department of Biomedical Imaging and Image guided Therapy Medical University of Vienna Vienna Austria; ^2^ Department of Radiology and Nuclear Medicine and Department of Nutrition and Movement Science Maastricht University Medical Center Maastricht The Netherlands; ^3^ Biomedical Research Consulting in Magnetic Resonance Spectroscopy Albuquerque New Mexico; ^4^ Department of Movement and Sports Sciences Ghent University Ghent Belgium; ^5^ Department of Diagnostics and Therapeutics University of Helsinki Helsinki Finland; ^6^ PeakAnalysts, Benenden Kent UK; ^7^ Section on Experimental Radiology, Department of Diagnostic and Interventional Radiology University Hospital Tübingen Tübingen Germany; ^8^ Institute for Diabetes Research and Metabolic Diseases (IDM) of the Helmholtz Center Munich at the University of Tübingen Tübingen Germany; ^9^ German Center for Diabetes Research (DZD) Tübingen Germany; ^10^ Departments of Radiology and Biomedical Research University and Inselspital Bern Switzerland

**Keywords:** acetylacarnitine, carnosine, deoxymyoglobine, intramyocellular lipids, lactate, magnetic resonance spectroscopy, physiology, skeletal muscle

## Abstract

^1^H‐MR spectroscopy of skeletal muscle provides insight into metabolism that is not available noninvasively by other methods. The recommendations given in this article are intended to guide those who have basic experience in general MRS to the special application of ^1^H‐MRS in skeletal muscle. The highly organized structure of skeletal muscle leads to effects that change spectral features far beyond simple peak heights, depending on the type and orientation of the muscle. Specific recommendations are given for the acquisition of three particular metabolites (intramyocellular lipids, carnosine and acetylcarnitine) and for preconditioning of experiments and instructions to study volunteers.

## Graphical abstract

The recommendations are intended to guide those who have basic experience in general MRS to the special application of 1H‐MRS in skeletal muscle. The structure of skeletal muscle leads to effects that change spectral features far beyond simple peak heights, depending on the type and orientation of the muscle. Specific advice is given for three particular metabolites (intramyocellular lipids, carnosine and acetylcarnitine) and for preconditioning of experiments and instructions to study volunteers.

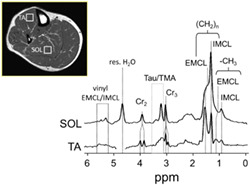

**Abbreviations used**
Cr: creatine
dMb: deoxymyoglobine
EMCL: extramyocellular lipids
IMCL: intramyocellular lipids
PCr: phosphocreatine
PRESS: point‐resolved spectroscopy
semi‐LASER: semi‐localized by adiabatic selective refocusing
STEAM: stimulated echo acquisition mode
TMA: trimethyl‐ammonium

## 1 INTRODUCTION

Skeletal muscle is the main tissue responsible for body posture and movement. To accomplish this function, it also demands a major part of whole‐body energy metabolism. Even at rest the musculature is responsible for ~ 30% of the metabolic rate of the human body. With this metabolic and physiologic function in focus and being accessible by early MR equipment, skeletal muscle was one of the first targets of examinations by in vivo ^31^PMR spectroscopy. In vivo ^1^H‐MRS examinations of human muscle followed with the advent of volume‐selective MRS methods. Over the years, in vivo ^1^H‐MRS has found its way into academic and clinical research in the fields of metabolism, diabetology, mitochondrial disorders, skeletal muscle dystrophy and sports physiology.

While general aspects of ^1^H‐MRS can be found either in textbooks or in other articles in this special issue on MRS recommendations, this article will emphasize the anatomical, morphological and biochemical properties of skeletal muscle and how these influence the acquisition, appearance and analysis of skeletal muscle ^1^H‐MR spectra, which differ considerably from the MR spectra of other organs. Rather than summarize a list of seminal papers that can be found in reviews that cover the field,^1^, ^2^, ^3^, ^4^, ^5^, ^6^ we will focus on methodological considerations. Specific recommendations for the measurement and assessment of intramyocellular lipids, acetylcarnitine and carnosine, three metabolites which are the focus of the majority of studies and publications in metabolic, physiologic and clinically oriented journals, are given. The final section tackles the measurement of deoxymyoglobin and lactate in ischemia and/or during anaerobic exercise, but without specific recommendations, as even recent papers do not answer all of the questions regarding the methodology of data acquisition and interpretation.

## 2 ANATOMICAL, FUNCTIONAL AND METABOLIC PROPERTIES OF SKELETAL MUSCLE

### 2.1 Structure and function

Musculature is tissue making up between 30% and 50% of body mass in healthy normal weight subjects.^7^ Muscles can be divided into three types: skeletal (or striated), cardiac and smooth. This article describes ^1^H‐MRS of skeletal muscle, which makes up the largest share of the body's muscle. This tissue is under conscious control and used for physical activity by articulating the joints of the skeleton.

**FIGURE 1:**
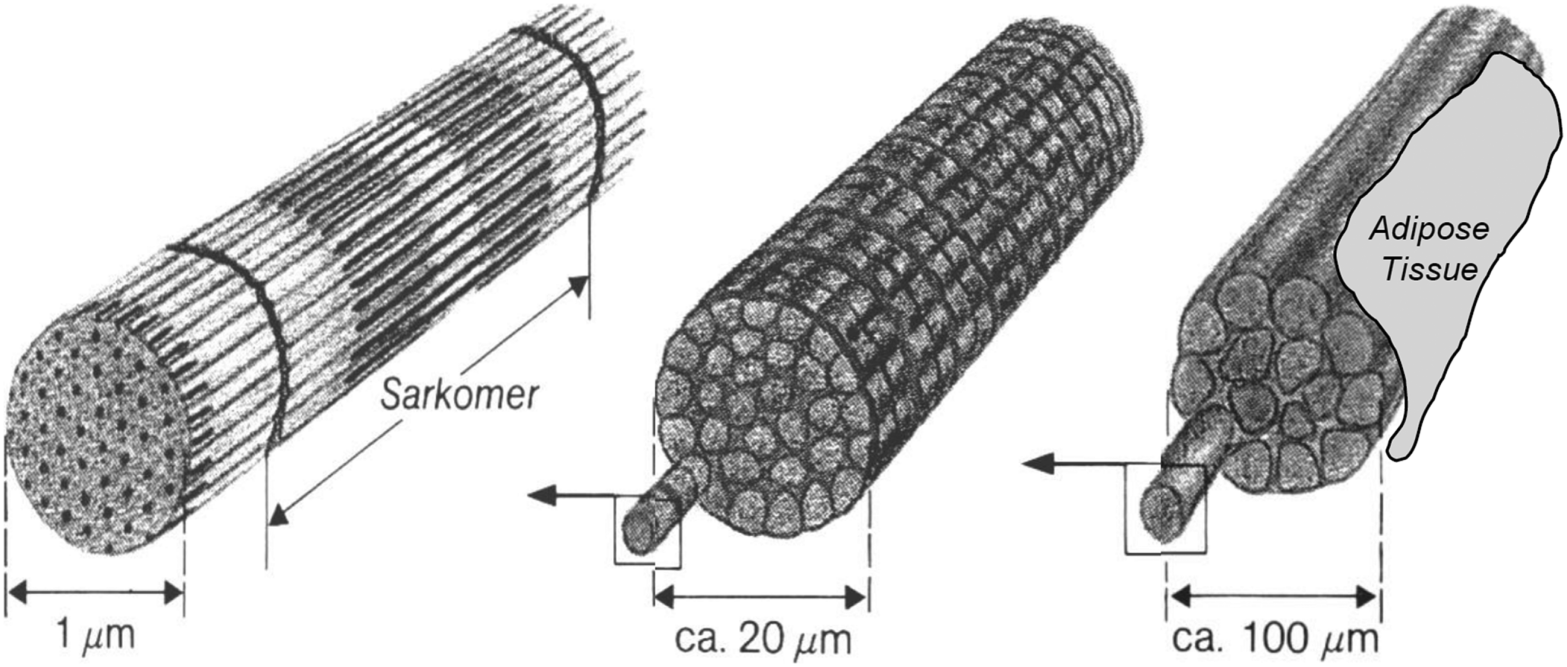
The inner structure of a skeletal muscle shows a high degree of geometrical order: one muscle (right) consists of many subunits called fascicles (middle), and each fascicle represents a bundle of many individual muscle fibers (left)

Muscle fibers consist of multinucleate cells, which were formed by fusing hundreds of myoblasts end‐to‐end. Each fiber is one elongated cell often extended for the length of the muscle. Microscopically, myocytes show a striated pattern that reflects the regular arrangement of protein structures in sarcomeres (basic functional units) within each cell. The inner structure of a skeletal muscle shows a high degree of geometrical order: one muscle consists of many subunits called fascicles, and each fascicle represents a bundle of many individual muscle fibers (Figure 1).^8^

Muscles produce force through the process of contraction of the sarcomeres (concentric motion). Tendons connect each end of the muscle to bone, which translates force production into limb movement. Stretching of a muscle (eccentric motion) is always passive and has to be performed by the effect of external forces or by contraction of an antagonistic muscle. Therefore, at least two muscles are working together for free movement of joints. Muscles in specific regions with (partly) common functions are often referred to as muscle groups (eg, calf muscles, quadriceps muscles) consisting of several muscles with different tendon insertions.

The force generated by the contractile elements of the myocytes has to be transferred to the tendon, which consists of mechanically resistant collagen fibers. For this purpose, collagen elements providing mechanical integrity are also present in the substructures of skeletal musculature: the endomysium is a thin layer of areolar connective tissue ensheathing each individual myocyte. This structure also contains capillaries and nerves. The perimysium is a thicker sheath of connective tissue grouping muscle fibers into the aforementioned fascicles, whereas a dense layer of connective tissue (termed epimysium) covers the entire skeletal muscle and forms the transition to tendons.^9^

Besides connective tissue, blood vessels and nerves, skeletal musculature also shows variable amounts and distribution of adipose tissue, which are mainly arranged in macroscopically visible septa along the muscle fiber bundles. This muscular adipose tissue consists of adipocytes and is often termed extramyocellular lipids (EMCL) in MR‐related literature, in contrast to clearly smaller lipid droplets inside myocytes, which are termed intramyocellular lipids (IMCL).^10^, ^11^

**FIGURE 2:**
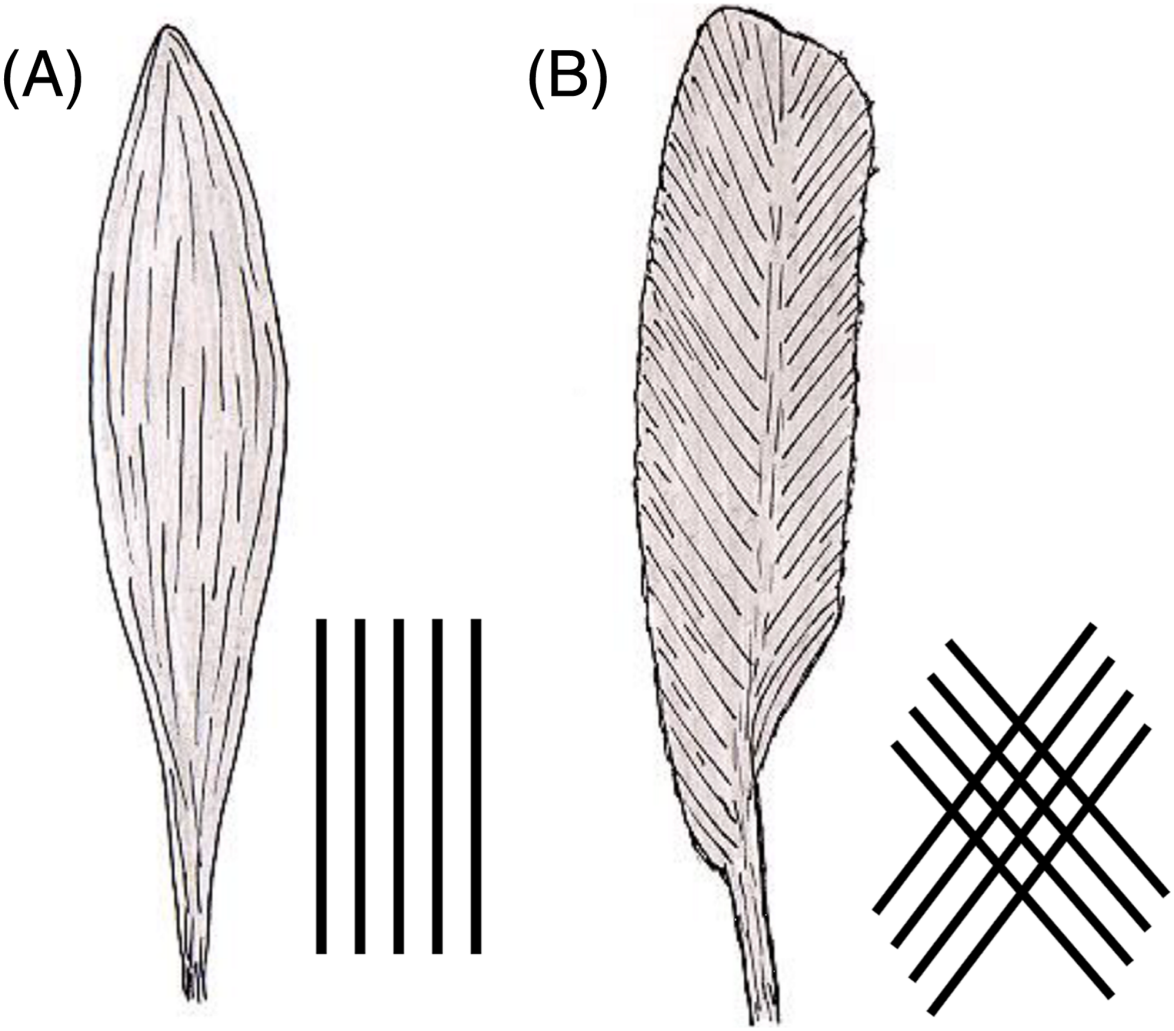
Typical shapes of skeletal muscle. In spindle‐shaped (fusiform) muscles (A; left), for example the tibialis anterior, fibers are oriented nearly parallel along the axis of the muscle. In feathered (pennate) muscles (B; right) such as the soleus muscle in the calf, fibers are arranged in a crossing pattern and the orientation of the fatty septa shows a distinct angle with respect to the orientation of the tendons

The arrangement of muscle fibers relative to the overall muscle orientation is a unique aspect of skeletal muscle physiology and determines both physiological action and features of ^1^H‐MR spectra. In spindle‐shaped (fusiform) muscles, for example the tibialis anterior, fibers are oriented nearly parallel along the axis of the muscle (Figure 2A). These muscles provide marked movability, but only limited force. By contrast, reduced movability but higher force is provided by feathered (pennate) muscles (Figure 2B), such as the soleus muscle in the calf. In feathered muscles, fibers present a crossing pattern and the orientation of the fatty septa shows a distinct angle with respect to the orientation of the tendons.

#### 2.1.1 Influence of skeletal muscle structure on ^1^H‐MR spectra

**FIGURE 3:**
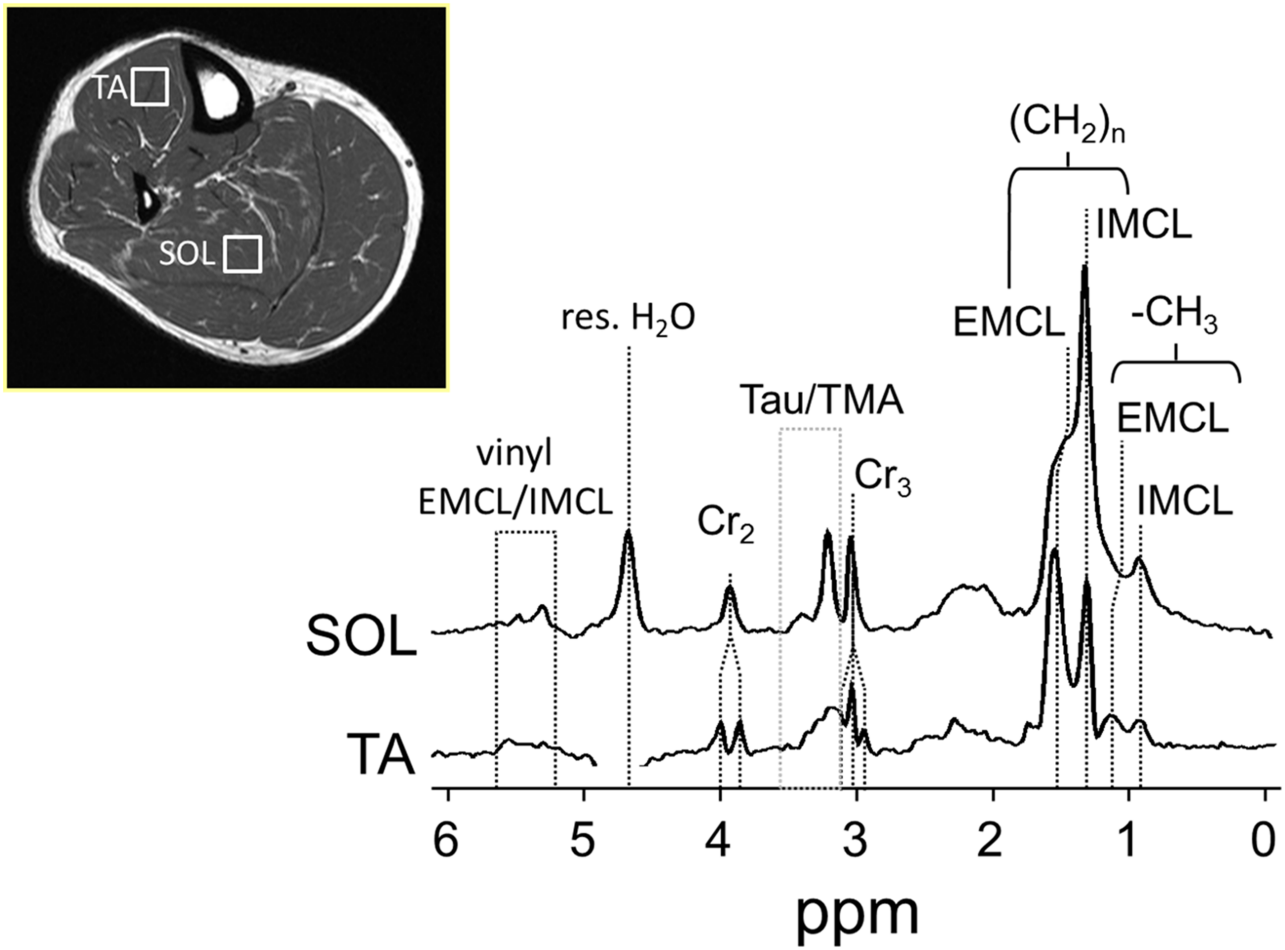
Representative ^1^H MR spectra of skeletal muscle recorded from the tibialis anterior (TA) and soleus muscle (SOL) at 3 T (PRESS, T_E_ = 30 ms). The different fiber orientation in each muscle group gives rise to different spectral appearance due to dipolar (creatine 3.05 and 3.9 ppm, taurine and TMA 3.2 ppm) or susceptibility (IMCL/EMCL 0.9‐1.5 ppm). Adapted with permission from Popadic Gacesa et al^6^

Proton MR spectra of skeletal muscle are quite different to those of other organs or those from aqueous solutions (Figure 3). The tissue microstructure complicates spectral features in at least three ways.

##### Susceptibility effects

Macroscopic and microscopic susceptibility differences cause local magnetic fields to vary strongly, such that lipid peaks of different compartments can be shifted in resonance frequency by up to ~ 0.2 ppm. The IMCL resonances are unaffected by these susceptibility effects while the EMCL resonances are shifted downfield relative to IMCL, depending on the pennation angle.^2^, ^10^, ^11^

##### Residual dipolar coupling

The high ordering of myofibrillar structures can restrict molecular motion, and for certain metabolites this leads to residual dipolar coupling effects that are not averaged out as in isotropic liquids, but generates line splitting, as in liquid crystals. In particular, the spectra of creatine/phosphocreatine (Cr/PCr), carnosine, taurine and lactate have been shown to feature residual dipolar couplings^12^ that are a function of the orientation of the muscle fibers within the region of interest to the external field. This may be due to the overall orientation of the muscle (eg, the angle between the long axis of the leg and B_0_), or to the pennation angle of the muscle fibers within a voxel. If the fiber orientation is not homogeneous and/or if the orientation changes during muscle strain, the spectrum may exhibit a mixture of patterns.

##### MR visibility of metabolites

Less understood is the fact that the MR visibility of some metabolites is not 100% and may depend on the muscle orientation. In particular, the trimethyl‐ammonium (TMA) group resonance at 3.2 ppm has proven to be only partially visible and to depend on orientation, possibly due to small variable dipolar couplings or orientation‐dependent transverse relaxation. In the same context, the TMA peak of acetylcarnitine (slightly upfield‐shifted compared with TMA of free carnitine) has been found to be represented by a broad signal pattern with possibly reduced visibility at 1.5 and 3 T,^13^ although it was reported to be fully visible at 7 T.^14^ Furthermore, free Cr seems to be invisible while PCr remains detectable^15^ under certain conditions. Whether this effect depends on external field strength, muscle or species type has so far remained open to debate^16^, ^17^ but may well be linked to multiple compartments of Cr/PCr that have been observed in other contexts. As a consequence of the limited visibility and orientation‐dependent resonance patterns, the use of the 3 ppm signal of total creatine (tCr) as standard for conversion to absolute concentration units is discouraged. Even although suggested by Kreis et al,^15^ the use of ^1^H MRS to observe phosphorylation kinetics by proton instead of phosphorous MRS has not been picked up by any group and needs verification in terms of advantages (no need for heteronuclear hardware, signal‐to‐noise ratio [SNR], localization options) and disadvantages (delicate measurement and evaluation given the proximity to the water resonance for the methylene resonance of phosphocreatine, open issues concerning visibilities).

### 2.2 Chemical composition and metabolic pathways accessible by ^1^H‐MRS

In skeletal muscle, the oxidation of fatty acids inside the mitochondria and glycolysis in the cytosol are the major pathways for producing the energy for muscle homeostasis and contraction. Fatty acids and carbohydrates can either be taken up from the bloodstream or obtained from storage forms (glycogen, EMCL, IMCL).

As described above, EMCL and IMCL represent two different forms of lipid storage with distinctly different physiological functions and ^1^H‐MR features. While the discrete localization and high concentration of EMCL makes MR imaging methods most appropriate, IMCL which are stored in droplets adjacent to mitochondria can be directly measured by ^1^H‐MRS.^1^, ^2^, ^10^, ^11^, ^18^

Lactate is a product of the anaerobic glycolytic process and it can also be detected by ^1^H‐MRS.^19^ The principal signal from lactate, a methyl group doublet at 1.35 ppm, is usually overlaid by lipid resonances, which hinder accurate detection. However, spectral editing is a method that exploits the ^1^H‐^1^H couplings within the molecule and enables unambiguous detection and quantification of lactate.^20^

Skeletal muscle proton spectra also feature the resonances of acetylcarnitine, carnosine and taurine.^21^ Acetylcarnitine is involved in the elimination of excessive Acetyl‐Co‐A. Buffering the acidic condition is one of the roles of carnosine and taurine is a product of cysteine and coenzyme‐A catalysis. Muscle fibers exist across a range of subtypes between slowly contracting, economical fibers with prevailing oxidative aerobic metabolism (type I fibers) to high‐speed fibers preferring carbohydrate fuels (type II fibers). Differences in the metabolism and functions between the fiber types can be accounted for by higher carnosine concentrations in muscles with a dominant proportion of fast fibers.^22^, ^23^

Muscle contraction and energy consumption results in increased oxygen demand and utilization that can also be monitored by surface coil‐localized ^1^H‐MRS deoxymyoglobin measurement.^24^ With this measurement and many other aspects, ^1^H‐MRS of skeletal muscle is complementary to ^31^P‐ and ^13^C‐MRS,^4^, ^25^, ^26^, ^27^, ^28^ which reveal major metabolites involved in energy storage and consumption, such as glycogen and/or high‐energy phosphorous compounds, which are not (or only indirectly) visible in ^1^H‐MRS.

## 3 ^1^H‐MRS OF SKELETAL MUSCLE

### 3.1 Common aspects of ^1^H‐MRS in different organs

Aspects of ^1^H‐MRS in skeletal muscle that are common to other organs will not be discussed in detail in this recommendation paper. These general considerations include single voxel and chemical shift imaging based on localization sequences, shimming, water suppression, motion artifacts due to blood flow or respiration, chemical shift displacement, outer volume suppression, spectral fitting, quantification, eddy current corrections, and other factors. More details can be found in excellent review articles and textbooks that describe the general basics of ^1^H‐MRS,^29^, ^30^, ^31^, ^32^ including other papers in this special issue.^33^, ^34^, ^35^, ^36^, ^37^, ^38^, ^39^ Only muscle‐specific facets of these issues that arise from the highly structured anatomy and the unique physiology and metabolism of skeletal muscle are discussed in the following sections.

### 3.2 Specific aspects of ^1^H‐MRS in skeletal muscle

Most published studies in humans use single voxel localization; fewer studies have introduced chemical shift imaging methods^2^, ^40^ or higher dimensional, correlated spectroscopic localization techniques.^41^ While chemical shift imaging methods offer the advantage of increased spatial coverage and resolution, the technical issues (longer acquisition times, acquisition‐type dependence on relaxation times, large datasets, water referencing, a requirement for sophisticated knowledge of techniques) limit their widespread application, and currently no specific recommendations can be formulated.

For single voxel spectroscopy, the voxel should be selected in homogeneous tissue, i.e., in one specific muscle. When muscles with different pennation angles contribute to the spectrum, a mixture of spectral features will complicate the fitting and interpretation. Since pennation angles vary even within the length of one muscle, localization should be chosen carefully, eg, avoiding regions with known fiber‐angle heterogeneity, and allowing exact repositioning in repeated examinations.

Furthermore, single voxel localization is inherently imperfect due to the slight transition bands in slice selection combined with a chemical shift displacement artefact (CSA). Therefore, whenever possible the voxel should be positioned in a location where it is surrounded by the same type of tissue. While this notion is valid for MRS in all organs, skeletal muscle shows particularly large differences in metabolite concentrations (eg, between EMCL and IMCL), which render even small contaminations from other compartments deleterious. On a typical 3 T system with slice selection using standard RF pulses, the location of the C2‐carnosine (at 8 ppm) or CH_3_‐lipid signal (at 0.9 ppm) may be displaced by up to 10%‐35% from that of the water resonance (at 4.7 ppm). Some MR systems illustrate the chemical shift displacement effect between water and the peak of interest within the user interface, which can assist in voxel placement. Ideally, we recommend acquiring individual spectra of metabolites and references with the peak of interest on‐resonance to eliminate chemical shift‐based spatial displacement. Finally, outer volume suppression slices may be used to eliminate signal coming from outside the selected volume, which would include signal shifted due to CSA as well as signal due to imperfection in slice selection, although this may increase the total measurement time.

Depending on the manufacturer of the MR system, a series of excellent shimming methods are available. However, shimming algorithms are often optimized for brain MRS and assume a relatively small voxel size (an edge length typically of 2 cm) and minimal lipid signals. These assumptions are often violated in muscle MRS and special care is advised if either very large (carnosine or acetylcarnitine) or very small (IMCL) voxels are selected, as discussed in the respective paragraphs.

While the selection of an appropriate coil is straightforward for brain MRS, it is more complicated in muscle MRS, where various combinations of transmit and receive coils are common. An excitation with the body coil has the advantage that the exciting RF field is homogeneous yet the pulse power and thus the bandwidth of the pulse is limited. Local volume coils, such as knee or extremity coils, are efficient for spectroscopy of the lower leg and knee‐adjacent portions of upper leg due to the relatively strong and homogeneous RF‐excitation. In combined multinuclear experiments in particular, two channel surface coils (eg, ^1^H/^31^P or ^1^H/^13^C) are used for excitation and reception of the ^1^H‐signal since no replacement of the coil is necessary between ^1^H and X‐nuclei acquisition^42^; however, since the excitation profile of a surface coil is inhomogeneous, special care must be taken to achieve an appropriate pulse angle. The choice of the receiving coil (volume coil vs. surface coil) will also introduce variations in sensitivity that can influence quantitation procedures.

**FIGURE 4:**
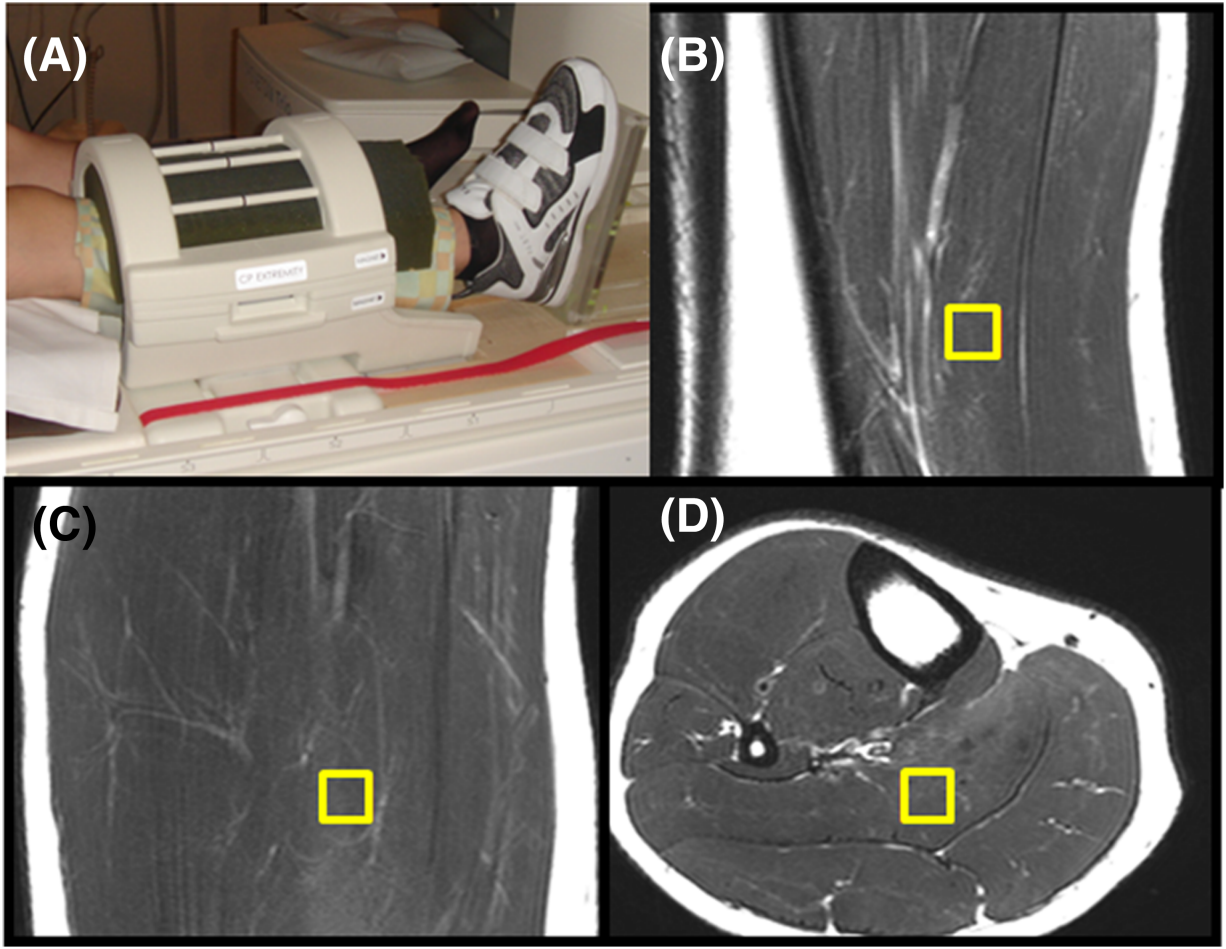
Fixation of leg and voxel selection. (A) Example of a tight yet still comfortable fixation of the leg: foam in the coil and a shoe fixed to the patient table restrict unwanted motion and in particular rotation. (B‐D) Cubical voxel selected in three orthogonal images of soleus, PRESS at 3 T

A major challenge of ^1^H‐MRS in skeletal muscle is elimination of leg motion. Restraining translational motion with foam or custom‐built devices usually works well. The prevention of rotation of the leg is more difficult, but can be accomplished by using a restraint (eg, a shoe) that is fixed to the patient table and controls alignment and rotation of the leg (Figure 4A).

### 3.3 Postprocessing and quantitation of ^1^H‐MR spectra from skeletal muscle

Most MR systems are equipped with vendor‐specific software for postprocessing; however, external software packages for offline postprocessing offer more advanced algorithms. Two of the most popular offline MRS postprocessing programs are jMRUI^43^, ^44^ and LCmodel,^45^ but many research groups also use custom‐written postprocessing routines. Many steps in postprocessing are generic (e.g., assessment of spectral quality and rejection of insufficient spectra, zero‐filling, apodization, time vs. frequency domain fitting, choice of Gaussian/Lorentzian/Voigt line shapes) and are not discussed in this article. However, aspects of prior knowledge and robust fitting constraints that are specific to IMCL, acetylcarnitine and carnosine are discussed in the metabolite‐specific paragraphs below.

To compare MR spectra from different acquisitions (eg, different volunteers), normalization of the spectra has to be performed. Metabolite content can be quantified with respect to a concentration reference. Using the tissue water signal as an internal concentration reference is the most common way to do this, however, it involves certain assumptions: (1) that tissue water content is not different between individuals; and (2) that it does not change with nutritional or hydration status. While these assumptions are relatively robust for homogeneous populations under standard conditions, they need to be considered for the particular study being conducted.^46^, ^47^

External concentration referencing is an alternative option that circumvents the assumptions of constant water content. General details of external referencing techniques can be found in the textbooks cited earlier, but the effects of coil loading/sensitivity, B_1_ field inhomogeneity and metabolite degradation within the external phantom should be considered. Metabolite‐specific aspects will be discussed in the relevant paragraphs.

Corrections for signal decay (T_2_) and signal saturation (T_1_) of metabolites and reference signals are necessary for an absolute quantitation, and these important parameters are summarized in Table 1 for skeletal muscle metabolites at different field strengths.^10^, ^14^, ^23^, ^48^, ^49^, ^50^, ^51^, ^52^ Appropriate T_1_ correction should be performed if T_R_ is less than 5‐fold of the longest T_1_ of interest, while a signal correction for T_2_ relaxation is generally necessary. Note that metabolite T_1_ and T_2_ relaxation times tend to be significantly longer in solution, e.g., in reference phantoms, and separate correction factors may be necessary.

**TABLE 1:** In vivo relaxation times of skeletal muscle metabolites*.* Data measured in soleus (Sol), gastrocnemius medialis (GM), tibialis anterior (TA) and vastus lateralis (VL). Data published in Schick et al,^10^ Krssak et al,^48^ Lindeboom et al,^49^ Baguet et al,^23^ Klepochova et al,^50^ Kukurova et al^51^ and Ren et al^14^, ^52^

	T_1_ [ms]			T_2_ [ms]		
	1.5 T	3 T	7 T	1.5 T	3 T	7 T
Water (4.7 ppm)	1100^10^	1380^48^ Sol/TA	1850^52^ GM/Sol 3340^51^ VL	50^10^	30	23^52^ GM/Sol 27^50^ VL 18 Sol
IMCL‐CH_3_ (0.88 ppm)	700^10^		1380^52^	200^10^		97^52^
EMCL‐CH_3_ (1.05 ppm)	400^10^		1200^52^	150^10^		74^52^
IMCL‐CH_2_ (1.28 ppm)	280^10^	370^48^ Sol 410^48^ TA	580^52^	85^10^	90^48^ Sol/TA	66^52^
EMCL‐CH_2_ (~ 1.45 ppm)	270^10^	370^48^Sol 420^48^ 48TA	570^52^	75^10^	77^48^ Sol/TA	51^52^
Acetylcarnitine (2.13 ppm)		2000^49^ VL	1807^50^ VL		265^49^ VL	130^50^ VL
Creatine‐CH_3_ (3.03 ppm)	1100^10^	1000^48^ Sol 1070 ^48^ TA	1380^14^ 1450^50^ VL	140^10^	135^48^ Sol/TA 162^49^ VL	103^14^ 166^50^ VL 132^50^Sol
Carnosine‐C_2_ (8.00 ppm)		1488^23^ Sol 1771^23^ GM	2000^51^ GM/Sol	100^10^	152^23^ Sol 106^23^ GM	96^51^ GM 81^51^ Sol

General recommendations for ^1^H MRS of skeletal muscle are listed in Table 2.

**TABLE 2:** General recommendations for ^1^H‐MRS of skeletal muscle

	It is strongly recommended that an expert with experience in in vivo MRS is involved. These recommendations emphasize only specific aspects of ^1^H‐MRS in skeletal muscle.
	All good practices in MRS have to be applied also in skeletal muscle, in particular quality issues of spectra.
Acquisition	Field strengths of 3 T and above are recommended for low concentration metabolites; however, especially for the IMCL measurement, 1.5 T can serve well.
	The choice of an appropriate coil is crucial. Small volume coils (eg, knee coils) are recommended.
	Elimination of leg motion is mandatory.
	In skeletal muscle, single voxel localization is recommended; chemical shift imaging methods have advantages yet should be applied only for specific aims and by experienced groups only.
	Voxel should contain one muscle (eg, soleus, gastrocnemius medialis, tibialis anterior) only.
	Shimming in skeletal muscle can be more complicated than in brain; very large voxel with fat signals and very small voxel size can cause difficulties. Full‐width‐at‐half‐maximum (FWHM) of water signal ≤20‐25 Hz (1.5 and 3 T) or 35‐40 Hz (7 T) in magnitude spectra should lead to sufficient spectral resolution.
	It is recommended to choose T_R_ ≥3‐fold the longest T_1_ of metabolites and reference.
	To avoid chemical shift displacement effects, on resonance acquisition is recommended for metabolites and reference signal in separate measurements.
Processing and Quantitation	Residual dipolar coupling, susceptibility effects and changes in MR visibility can strongly change the ^1^H‐MR spectral pattern in skeletal muscle and need to be considered.
	Internal and external referencing for the calculation of absolute concentrations have specific advantages and disadvantages that need to be considered, as in other organs.
	Correction for T_2_ decay is necessary for metabolites and reference. As long as no variations of T_2_ are expected, literature values of T_2_ are appropriate.
	Correction for T_1_ saturation is necessary if T_R_ was significantly shorter as 5‐fold the longest T_1_ of metabolites and reference.

## 4 ^1^H‐MRS OF IMCL

### 4.1 Introduction

**FIGURE 5:**
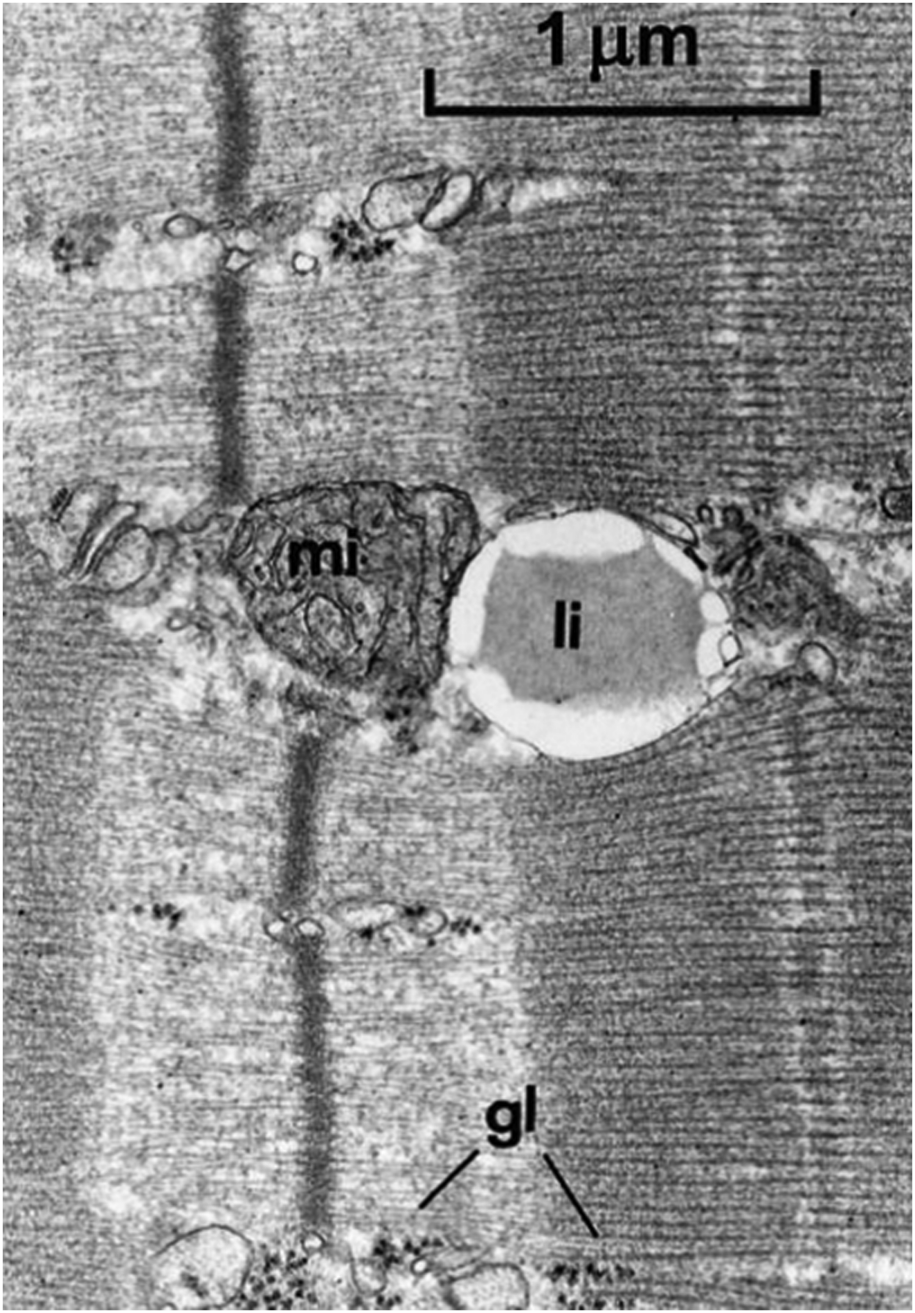
IMCL droplets (li‐lipids, IMCL) in an electron microscopic image from skeletal muscle. Spherical droplets are close to mitochondria (mi) indicating the metabolically active role of IMCL. Adapted with permission from Boesch et al^2^

**FIGURE 6:**
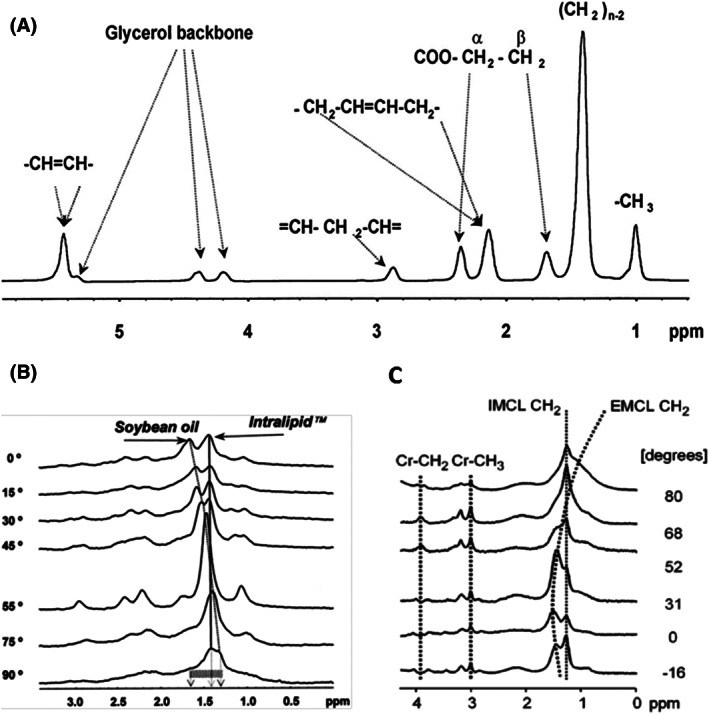
^1^H MRS spectra of lipids. (A) High‐resolution proton spectrum of soybean oil acquired at 7 T. Resonances are assigned to protons of fatty acid chains and glycerol as indicated. (B) Orientation dependence in a model of intralipid (spherical vesicles) and soy bean oil (cylindrical‐shaped vial) acquired at 4.7 T. Relative angle in respect to B_0_ magnetic field is given in degrees. (C) Orientation dependence in human muscle (m. tibialis anterior), acquired on 1.5 T (PRESS, TE 20 ms, TR 3000 ms); the position of EMCL methylene resonance is dependent on the angle between the muscle and B_0_ magnetic field, which is given in degrees. Adapted with permission adapted from Szczepaniak et al^18^ (A, B) and Boesch et al^11^ (C)

Two types of lipid pools in skeletal muscle can be distinguished by ^1^H‐MRS noninvasively.^10^, ^11^ The reason for the frequency shifts are susceptibility effects, which shift EMCL in subcutaneous fat and along fasciae, while IMCL in lipid droplets (Figure 5) remain at the frequency position, which is known from solutions due to the spherical shape.^11^ In contrast to the biochemical literature, where the term intramyocellular triglyceride is often used, the notation EMCL/IMCL recommended in MRS uses the overarching term lipids to emphasize that in vivo MRS cannot distinguish between free fatty acids, triglycerides and sufficiently mobile amphiphilic lipids. Rotation experiments in vivo^11^ and in model solutions of intralipid/soybean oil^18^ unraveled the susceptibility nature of the observed frequency shifts (Figure 6). This orientation dependence affects skeletal muscle in two ways, either when the muscle is rotated against the static magnetic field or when the specific muscle fibers are tilted within the muscle (pennation angle). In both cases, the separation of IMCL and EMCL resonances is affected (Figures 3 and 6).

The interest in studying skeletal muscle lipid content via localized ^1^H‐MRS in vivo was sparked by the demonstration that the proximity of IMCL is a highly active energy storage form that can be used and replenished within short time periods in healthy subjects,^11^, ^53^ while insulin‐resistant patients have constantly increased IMCL levels.^54^, ^55^, ^56^, ^57^ It was shown that a saddle‐shaped correlation exists between insulin resistance, VO_2max_ and IMCL levels at rest.^58^

### 4.2 Detection and quantification of IMCL

The selection of a specific muscle is mainly determined by the physiological aim of a study (e.g., comparison with biopsy data, dominant fiber types); however, the selection should, whenever possible, also take into account that some muscles are more suited for ^1^H‐MRS. Due to the orientation dependence of the EMCL/IMCL separation, the best separation of signals will be achieved in fusiform muscles with a uniform fiber orientation along the axis of the muscle, eg, m. tibialis anterior or m. vastus intermedius. If the study design involves feathered muscles with varying fiber orientations (eg, m. soleus), the separation of EMCL/IMCL is smaller and thus contamination of the IMCL resonance by EMCL is more likely.

After selecting the specific muscle, extended image series need to be acquired, particularly if repeated measurements require accurate repositioning. High resolution T_1_‐ or T_2_‐weighted orthogonal, contiguous axial or 3D images are mandatory (Figure 4B‐D). In addition, using a relatively small bandwidth results in a (usually unwanted) chemical shift artifact that can help to identify small fat inclusions along fasciae. Exact repositioning in the transversal image plane is usually easier than along the muscle, where fewer landmarks can be found. It is recommended to compare the high‐resolution axial series in repeated measurements and to identify landmarks, such as the bifurcations of vessels or peculiar lipid formations in both series, to calculate an inferior‐superior displacement between subsequent series. If permitted by the field of view, distant anatomical marks, such as the patella or the tibia plateau, are helpful for detecting a shift between series.

**FIGURE 7:**
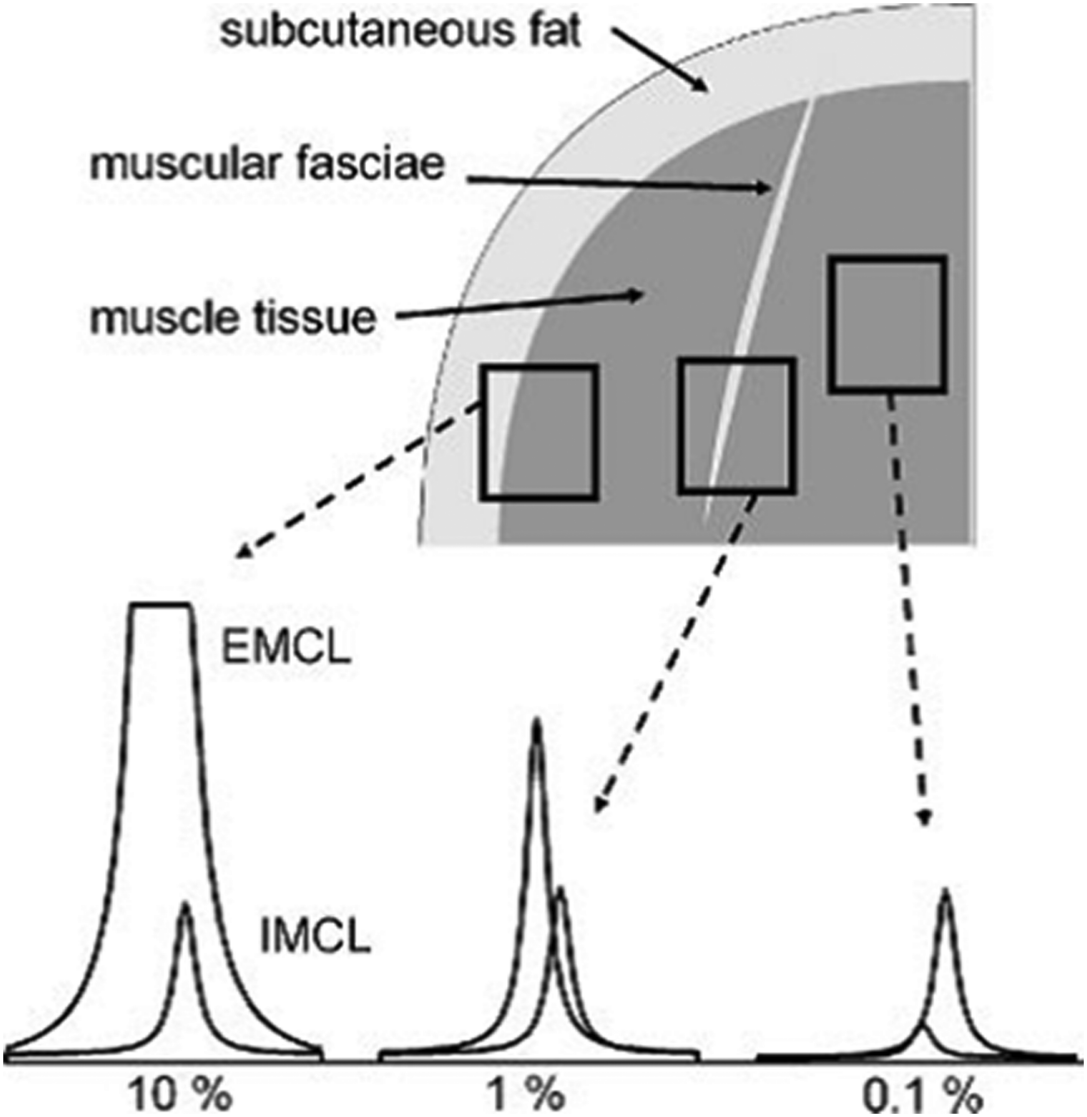
Influence of appropriate voxel position on spectral appearance in the lipid region. Careful placement of the voxel in muscle excluding of fascia results in spectra with a slight EMCL contamination (right). If such a fasciae, which may be hardly visible in a common MR image, is included in the voxel, the IMCL signal may become dominated by a large contamination from EMCL (middle). As soon as adipose tissue is included, eg, subcutaneous fat at the border of a voxel, the IMCL signals are no longer visible (left). Adapted with permission from Boesch et al^2^

The positioning of the voxel is a crucial part of the quality of ^1^H‐MRS of IMCL since the adjacent signal from EMCL is usually significantly stronger. Therefore, regardless of the orientation of the leg relative to the direction of the magnetic field, if EMCL content in the voxel is high, then the EMCL signal covers the IMCL signal and a reliable fitting of IMCL becomes increasingly difficult (Figure 7).^2^ In general, the voxel size for IMCL acquisition should be as small as SNR allows, ~ 10 mm x 10 mm in the transversal image plane. If necessary, the voxel size should be increased preferentially along the muscle axis (typically to less than 20 mm). For consistent quantitative results, the voxel size should be the same for all study subjects at all study time points, allowing only small adaptions for exclusion of obvious EMCL contributions and deviating muscle size.

As mentioned above, shimming procedures from the manufacturers are often optimized for brain spectra and might fail on small voxels, which are preferred to avoid EMCL contamination. Constraints on minimal shimming volumes should be obtained from the manufacturer and, if necessary, the shimming volume should be placed asymmetrically to avoid larger EMCL depots, which might confuse phase‐sensitive algorithms. To achieve a good shim, the voxel should be placed as far away as possible from bones, adipose tissue, blood vessels and the connective tissue. It is also recommended to prove the quality of the shim prior to data acquisition by inspection of the localized water signal, and to perform a manual readjust whenever needed (if feasible).

Quantitative measurement of EMCL in musculature is hampered by its inhomogeneous distribution. Single voxel ^1^H‐MRS is inappropriate for EMCL since even small voxel shifts lead to almost arbitrary variations. For a quantification of EMCL, fat‐selective imaging approaches^1^ should be considered.

While the choice of T_E_ and T_R_ is dictated by the T_1_ and T_2_ of lipids and water (Table 1), as described in general recommendations to use rather short T_E_ (<30‐40 ms) and moderate T_R_ of ≥2000 ms, differences between T_2_ of EMCL and IMCL can be exploited through long T_E_ acquisition approaches (T_E_ ≥ 150 ms) for improvement of line splitting between EMCL and IMCL peaks.^52^, ^59^ However, such spectra are heavily T_2_‐weighted and even exact knowledge of T_2_ relaxation times cannot avoid quantification errors.

Spectral processing for IMCL quantitation starts with visual inspection of the spectra. Several fitting procedures are available, yet it cannot be sufficiently emphasized that clearly visible line splitting of IMCL and EMCL in the methyl and methylene region (0.9‐1.5 ppm) is necessary for reliable fitting. If the EMCL CH_2_ signal is strong, small uncertainties in the EMCL linewidth and shape can substantially influence the adjacent IMCL‐CH_2_ signal. In general, strong prior knowledge input into the fitting algorithms is recommended. This includes fixed or at least related linewidths of the contributing peaks. While IMCL peaks can be modeled more easily, EMCL CH_2_ linewidth and position can change with fiber orientation. In general, a mixture of Lorentz and Gauss lines (resulting in Voigt lines) is preferable.

LCModel^45^ provides a commercially available algorithm with IMCL and EMCL signals included in the basis dataset. If jMRUI^43^, ^44^ or fitting algorithms embedded in the scanner software are used, then an extended set of prior knowledge is required to make IMCL/EMCL separation robust. Suggestions for prior knowledge also applicable in other frequency‐ or time‐domain approaches, and calculations of correction factors accounting for different reference signal (water, Cr, bone marrow lipids) and average fatty acid chain length and chemical bond saturation, can be found in review article.^2^

Reproducibility on measurements of IMCL has been assessed in studies showing intra‐day variations of less than 12% for tibialis anterior and soleus muscle,^59^, ^60^, ^61^ which are low compared with inter‐individual differences (several 100%).

Most fitting and quantification approaches^11^ use an average composition of lipids in IMCL to estimate the chain length and unsaturation (ie, the number of CH_2_ per fatty acid chain in the methylene peak). This is reasonable yet not fully accurate since it has been shown that lipids in other adipose tissue compartments can change saturation and chain length with diet^62^; more recently, similar variations between groups have been reported for IMCL during fasting protocol.^63^ While it is still reasonable to assume that large changes in the CH_2_ signal of IMCL are due to changes in IMCL concentration, it is important to be aware of this simplification since CH_2_ signal intensity could also change with a reduction/increase of CH_2_ per fatty acid chain due to a shorter/longer average chain length or un/saturation.

### 4.3 Physiological considerations and measurement preconditioning

Concentrations of IMCL in skeletal muscle are affected by the long‐term metabolic phenotype (insulin sensitivity, mitochondrial activity, training status) and also by short‐term influences such as exercise and diet.^64^Exercise stimulates fat oxidation and therefore results in depletion of IMCL during a single bout of submaximal exercise^65^, ^66^, ^67^, ^68^ or even moderate hiking.^11^, ^69^ The utilization of IMCL depends on the exercise duration and intensity; low‐intensity submaximal schemes use relatively more lipid, and high‐intensity bouts use more carbohydrates.^70^, ^71^ Whereas exercise stimulates fat oxidation in active muscle, plasma‐free fatty acid concentrations also increase during exercise, which may lead to a concomitant increase in IMCL content in nonexercising muscles, eg, in the arms during a leg workload.^72^Another well‐documented modulator of IMCL is a fat‐rich diet, which generally results in elevated IMCL content, even after just a few days.^65^, ^73^, ^74^, ^75^ Similarly, an elevation of plasma fatty acids, either after prolonged fasting due to the stimulation of lipolysis in adipose tissue^76^, ^77^ or due to intravenous lipid infusion‐increased exposure of muscle to free fatty acids,^75^, ^78^ is followed by pronounced elevations of IMCL content.

Other factors influencing substrate oxidation may have an effect on IMCL content, although some of them have not been thoroughly investigated. In contrast to starvation over several days, which increases IMCL levels, IMCL levels are reduced in the evening with a low‐caloric/low‐fat diet, especially if combined with physical activity.^67^ Other factors that affect substrate oxidation, fatty acid uptake and lipolysis, including hormonal influences, have yet to be studied extensively.

Because of the differences in muscle fiber composition and the different loading of various muscle groups in daily life and exercise challenges, it is not surprising that IMCL levels and repletion/depletion vary between different muscles.^2^, ^75^

Studies of resting IMCL levels require careful standardization as short‐term variations in IMCL content due to confounding factors (eg, diet or activity level) may be of similar magnitude to the effect being investigated (e.g., a cross‐sectional comparison of metabolic phenotypes or the effect of an intervention). It is therefore vital that such factors are accounted for in the study design.

Strenuous exercise should be omitted in the 2 days preceding IMCL investigation, and an energy‐balanced diet is recommended for 3 days before the examination. MRS measurements should be performed at the same time of day to avoid diurnal changes in activity and thus lipid content or the effects of daily behavior.

Special care is warranted if groups with completely different lifestyles are compared since the adherence to the standardization protocol may differ: for example, an instruction of "no vigorous physical activity" might not prevent athletes using their bikes over long distances to come to the MR center, while less trained subjects might refrain from any extra movement. A comparison of resting levels between such groups needs detailed instructions and a diary or monitoring devices for activity tracking. Since preparation of the volunteers and accurate acquisition/analysis of IMCL has to consider many details, it is recommended—as for other nonroutine MRS applications—to seek technical advice from experienced researchers.

Specific recommendations for ^1^H MRS of IMCL are listed in Table 3.

**TABLE 3:** Recommendations for ^1^H MRS of intramyocellular lipids (IMCL)

Acquisition	Whenever the study aim allows, fusiform muscles with an orientation along the static magnetic field are recommended to increase EMCL/IMCL separation.
	Extended high‐resolution imaging series are mandatory to find an optimal voxel position; in particular, preintervention and postintervention studies require careful repositioning.
	Positioning of the small, anisotropic voxel (eg, 10 mm x 10 mm x 18 mm along muscle) must, whenever possible, avoid visible EMCL along fasciae.
	Manufacturer‐provided shimming routines might be optimized for brain and fail in muscle. Shimming volume, localization and algorithms need to be adapted.
	Single‐voxel MRS is inappropriate for a determination of EMCL levels, which requires fat‐selective imaging techniques
Processing and /Quantitation	Clearly visible spectral separation of EMCL/IMCL methylene signals is a prerequisite for a robust fitting procedure, either with commercially available or self‐tailored fitting routines.
	Self‐tailored fitting routines (e.g., jMRUI) require relatively strong prior knowledge boundaries to be sufficiently robust.
Physiology and Preconditioning	IMCL levels are highly dynamic (exercise, diet) and also influenced by long‐term effects (eg, lifestyle, insulin sensitivity) and thus, careful preconditioning of subjects is crucial.
	Comparison of resting IMCL levels in groups with different lifestyle easily introduces biases between groups; an adapted instruction and supervision regrading diet and physical activity of the groups is mandatory.
	Large variations in macronutrient composition should be avoided for 2 weeks and feeding to maintain energy balance should be ensured in the last 3 days prior to IMCL investigation. Ideally, the diet should be provided, alternatively, a diary can help to follow the dietary habits of the subjects.
	Exercise‐induced variation of IMCL levels are influenced by intensity (40%‐60% of VO_2_max), duration (≥60 min), muscle involved and fed/fasting state.
	Combination of light‐to‐moderate exercise and caloric restriction over several days might help to deplete IMCL in patient groups with limited exercise capacity.
	Replenishment of IMCL levels takes several hours to days and depends on the type of diet and the observed muscle group.

## 5 ^1^H‐MRS OF CARNOSINE

### 5.1 Introduction

Carnosine (beta‐alanyl‐L‐histidine or (E)‐N‐(3‐Amino‐1‐hydroxypropylidene)‐L‐histidine) is a dipeptide composed of the proteinogenic amino acid histidine and the beta‐amino acid beta‐alanine. It is typically present in high concentrations in mammalian excitable tissues, and most prominently in skeletal muscle.^79^ Although full insight into the physiological roles of carnosine in muscle remains incomplete, the proton‐buffering capacity of the imidazole moiety of histidine (pKa of 6.72) dictates a likely involvement in acid‐base regulation during anaerobic energy delivery for intense muscle contractions.^80^ Additionally, carnosine has been presumed to serve a role conferring calcium sensitivity to the myofibrillar contractile apparatus modulating calcium release and reuptake during excitation‐contraction coupling, especially in the fatigued state.^81^ Further roles of carnosine may relate to its antioxidant capacity and reactive carbonyl‐sequestering capacity, but these contributions to skeletal muscle homeostasis are still under investigation.^79^

### 5.2 Detection and quantification of carnosine

**FIGURE 8:**
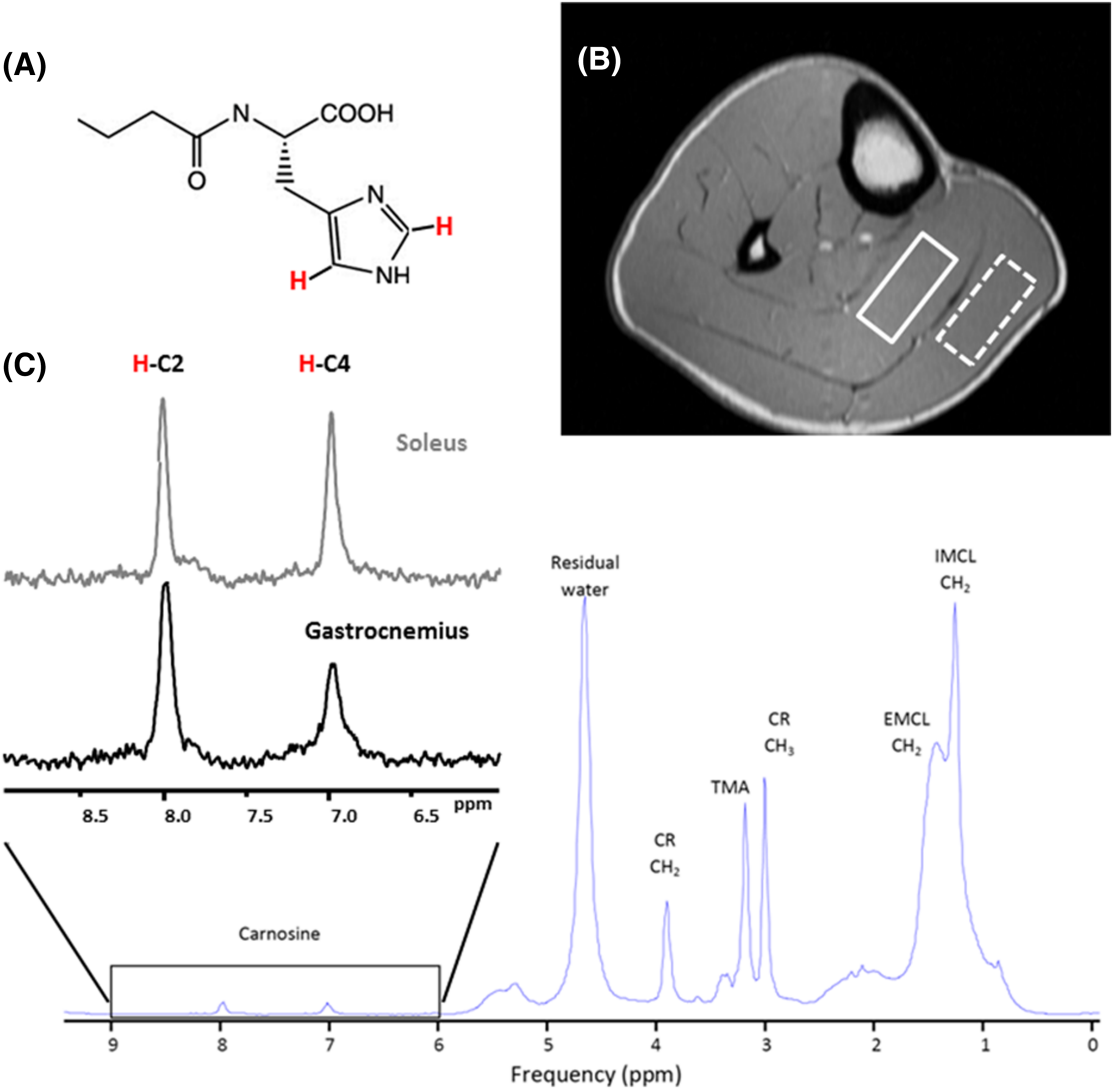
^1^H spectrum of the carnosine. (A) Chemical structure of carnosine. (B) Positioning the voxel for ^1^H MRS carnosine acquisition in soleus (full line box) and gastrocnemius (dashed line box) at 3 T using a PRESS sequence and a 15.5 cm diameter, 15‐channel, Tx/Rx extremity coil. Voxel size: 40 mm x 12 mm x 30 mm; TR: 2000 ms; TE: 30ms; 128 averages. Imidazole resonances acquired from the soleus (lower panel, C). The inherent linewidth of the H‐C2‐carnosine peak is ~ 6.5 Hz. Left insert (C) compares the ^1^H spectrum of the H‐C_2_ and H‐C_4_‐carnosine imidazole resonances acquired from the soleus and gastrocnemius at 3 T. The effects of residual dipolar coupling are negligible in soleus due to optimal muscle fiber alignment, resulting in equivalence of the H‐C2 and H‐C4‐carnosine resonances. In gastrocnemius, fiber‐alignment is nonoptimal and the H‐C4‐carnosine peak exhibits dipolar‐coupling induced splitting, which, along with muscle‐specific differences in T_2_, contribute to the signal mismatch

Initial studies demonstrated that the protons attached to the C2 and C4 carbons of the imidazole ring of carnosine (the aromatic structure of the histidine side‐chain) are visible in the (less crowded) downfield region of ^1^H spectra,^82^, ^83^ with chemical shifts at ~ 8 and 7 ppm, respectively (Figure 8).

The resonance frequencies of the H‐C2 and H‐C4 protons of carnosine are pH‐sensitive, and early studies focused on the use of carnosine as a marker of intracellular pH. Muscle contractions leading to the development of intramyocellular acidosis were accompanied by a shift of the H‐C2 carnosine peak from 8.0 to 8.3 ppm, corresponding to a physiological decline in tissue pH from 7 to 6.3.^83^ The H‐C2 carnosine resonance could therefore provide a ^1^H‐based alternative to the more common ^31^P‐MRS technique, which utilizes the chemical shift of inorganic phosphate (Pi) as a biomarker of pH during contractions. This permits simultaneous monitoring of pH and lactate in contracting muscle^84^ and avoids the significant issue of the ^31^P‐MRS approach, where the Pi peak amplitude depends on the history fiber activation. Kukurova et al^51^ recently reported a study at 7 T where the H‐C2 carnosine resonance of gastrocnemius muscles split following fatiguing exercise, which most likely demonstrates the existence of two muscle compartments: a fast‐twitch fiber pool which undergoes acidosis (and thus a downfield shift of H‐C2 peak), and a slow‐twitch fiber pool which can maintain pH homeostasis. Interestingly, muscle carnosine has also been proposed as a biomarker of intracellular pH in clinical applications such as Duchenne muscular dystrophy.^85^

Several strategies have been adopted to account for the lower molar concentration of carnosine compared with Cr/lipid to yield a sufficiently high SNR. Although carnosine can be detected at 1.5 T,^86^ the majority of studies have been performed at 3 T^87^, ^88^ or even higher field strengths.^51^, ^83^ The voxel volume is usually expanded to 5‐15 ml, significantly larger than that typically used for Cr and IMCL. In order to fit such a large voxel within a single muscle (eg, gastrocnemius), the voxel is usually extended along the length of the muscle; careful positioning is therefore required to ensure that the region of interest remains within the targeted muscle. With a relatively large acquisition volume, which lies in close proximity to muscle fascia and deposits of EMCL, shimming algorithms should be carefully monitored to ensure optimal performance; nonstandard procedures (eg, incorporating lipid suppression) may need to be adopted. In order to maximize detection sensitivity, a PRESS or semi‐LASER sequence should be adopted, although STEAM has also been used.^86^

Based on the T_1_ and T_2_ relaxation times for the 8 ppm resonance of carnosine (Table 1), short T_E_ (≤30‐40 ms) sequences with a T_R_ of ≥2000 ms, a relatively high number of spectral transients acquired (128‐256) and relatively long acquisition times (4‐10 minutes) are usually required.

In general, the carnosine imidazole resonances are well separated from the peaks of other major metabolites, and simple peak integration following local baseline correction across a well‐defined frequency range or standard line‐fitting routines should work. The H‐C2 peak is less susceptible to the effects of residual dipolar coupling and has a longer apparent T_2_ than the H‐C4 peak and is thus preferred for quantification (Figure 8B). We estimate that the contribution of dipolar‐coupled satellite peaks to the overall H‐C2 carnosine signal is <9% with careful limb positioning and voxel placement.^89^ However, for accurate quantification, these satellite peaks should be included in the spectral analysis.

Neighboring resonances from the imidazole group of histamine/histidine (at ~ 7.8 ppm), and the adenosine moiety of ATP (at 8.2 and 8.5 ppm), may be detectable,^90^ but histidine concentrations are an order of magnitude lower than carnosine and at 3 T frequency dispersion is sufficient so that these do not overlap with H‐C2 carnosine. The imidazole ring NH proton of carnosine has a putative chemical shift of ~ 8.1 ppm but in vivo this proton undergoes rapid exchange and is likely to be nonvisible.

In m. soleus, where muscle‐fiber orientations are optimal and residual dipolar coupling negligible, there is equivalence between the observed H‐C2 and H‐C4 carnosine peak areas. However, in other muscle groups (e.g., m. gastrocnemius), the H‐C2 carnosine peak is evidently larger than the H‐C4 peak. This signal mismatch is likely attributable to residual dipolar coupling and differences in T_2_ between the H‐C2 and H‐C4 peaks and the presence of a contaminating species at ~ 8.0 ppm, eg, the amide proton of the peptide bond between the histidine and beta‐alanine residues of carnosine.^89^ Additional studies are required to fully exclude a contribution from the amide proton to the nominal H‐C2 carnosine peak in muscle in vivo.

Quantification of carnosine from ^1^H‐MRS data (e.g., expressed as mmol/L (muscle) has typically used water as an internal concentration reference (as discussed in section 3.3). While this approach appears reliable for homogeneous subject cohorts, the inherent assumption of constant water content and its relaxation times may fail in elderly or obese subjects.^46^, ^47^ Although more challenging experimentally, quantification using an external reference phantom can be performed.^29^ Similarly, T_2_ relaxation times of both water and carnosine may vary across disparate subject cohorts. In these instances, metabolite T_2_ relaxation times of the specific study population should be measured and preferably used in conjunction with short T_E_ acquisitions.^91^

### 5.3 Physiological considerations for study design

Muscle carnosine content displays a remarkably high inter‐individual variability in humans, which can be explained by several determinants.^92^ Concentrations are ~ 20%‐30% higher in men than women at adulthood, but this sexual dimorphism is not observed prepuberty.^93^ In males, there is a clear increase in muscle carnosine concentrations from childhood to adulthood. Following adolescence, there is a gradual decline in both sexes with advancing age.^93^ However, despite accounting for age and sex, a considerable inter‐individual variability in muscle carnosine content remains, which is best explained by the muscle fiber type composition. The concentration of carnosine in fast‐twitch muscle fibers is approximately double that in slow‐twitch fibers.^22^ Therefore, individuals with a dominant proportion of fast fibers typically display high total muscle carnosine content.^23^

Intra‐individual differences between muscle groups are observed, again principally dependent on the muscle fiber type composition: the postural soleus muscle (dominant in slow‐twitch fibers) has consistently been found to have 20%‐30% lower concentrations than the more phasic (and fast‐twitch) gastrocnemius muscle.^87^ Given these intra‐individual differences between muscle groups, it is strongly advised to perform spectroscopy on a single muscle only. Muscle carnosine concentrations determined using ^1^H‐MRS techniques fall in the range of 4.9‐7.3 mmol/L (muscle), depending on the muscle type and population under study, consistent with the biochemical analysis of human muscle biopsies (20‐30 mmol/kg dry weight).^22^

Chronic oral beta‐alanine supplementation can elevate muscle carnosine considerably,^22^ which is accompanied by improved muscle function and exercise capacity,^22^, ^87^ in particular for high‐intensity exercise bouts.^94^ In parallel to its growing popularity in sport and exercise science,^95^ there is also increased interest in the clinical and pathophysiological relevance of muscle carnosine.^96^ Cross‐sectional studies found associations between muscle carnosine and insulin resistance^97^ and declines have been suggested in type 2 diabetes, multiple sclerosis and aging. These factors need to be considered if group comparisons are studied.

Specific recommendations for ^1^H MRS of carnosine are listed in Table 4.

**TABLE 4:** Recommendations for ^1^H MRS of carnosine

Acquisition	Different muscle groups exhibit significantly different carnosine concentrations; a single muscle group should be examined.
	Relatively large voxels (5 to 15 mL) are required for optimal SNR. A nonisotropic orientation may be required.
	Magnetic field strengths ≥ 3 T are preferred. For optimal SNR, a PRESS acquisition with short T_E_ (≤30 ms), long TR (≥2000 ms) and 128‐256 acquisitions is recommended.
	The effects of residual dipolar coupling effects can be minimized with appropriate limb positioning and voxel placement and are negligible in soleus.
Processing and /Quantitation	Other muscle groups than soleus can be studied but the effects of dipolar coupling must be considered. If present, dipolar‐coupled satellite peaks should be incorporated into the spectral analysis.
	The H‐C2‐resonance at 8.0 ppm is preferred for quantitation due to smaller residual dipolar coupling effects and longer T_2_ values than the H‐C4‐peak at 7.0 ppm.
	The carnosine resonances are pH sensitive – this effect should be considered during spectral analysis if pH changes are expected.
	Absolute concentrations of carnosine can be estimated in homogeneous cohorts using internal referencing (water). External referencing may be preferable if water content varies due to age, obesity or hydration status.
Physiology and /Preconditioning	Muscle carnosine concentrations are dependent on gender, age and training status. Careful and unbiased selection of study volunteers is recommended.
	Carnosine concentrations can be dependent on specific dietary/nutritional status (standardization or at least documentation of diet prior to the study).

## 6 ^1^H‐MRS OF ACETYLCARNITINE

### 6.1 Introduction

Acetylcarnitine (3‐Acetoxy‐4‐(trimethylammonio)butanoate) is synthesized in muscle from carnitine and acetyl‐CoA by the enzyme carnitine acetyltransferase (CrAT) in situations where mitochondrial acetyl‐CoA is abundant and exceeds the usage of it by the tricarboxylic acid (TCA) cycle. A single bout of exercise, for example, leads to a rapid and prominent increase of acetylcarnitine levels in vivo. This exercise‐induced elevation could be used to observe the acetylcarnitine resonances, even when overlapping with other metabolites,^13^ by subtracting pre‐ and postexercise spectra from skeletal muscle. In the studies at 1.5 T, the difference spectrum reveals a narrow, well‐resolved singlet at 2.13 ppm, as well as a signal at 3.17 ppm^13^, ^98^ in the TMA region. With the introduction of high field MR systems (e.g., 7 T), it became possible to resolve these resonances after exercise without subtraction.^14^

More recently, it has become apparent that the formation of acetylcarnitine might play an essential role in maintaining metabolic flexibility (e.g., adjustment of mitochondrial fuel selection in response to metabolic cues).^99^ The noninvasive detection of acetylcarnitine thus enables us to gain new insights into mitochondrial intermediary metabolism and to better understand the role of acetylcarnitine formation in vivo under various conditions. To investigate this further, the quantification of exercise‐induced differences in acetylcarnitine was insufficient and the quantification of acetylcarnitine concentrations in the resting state is required.

### 6.2 Detection and quantification of acetylcarnitine

**FIGURE 9:**
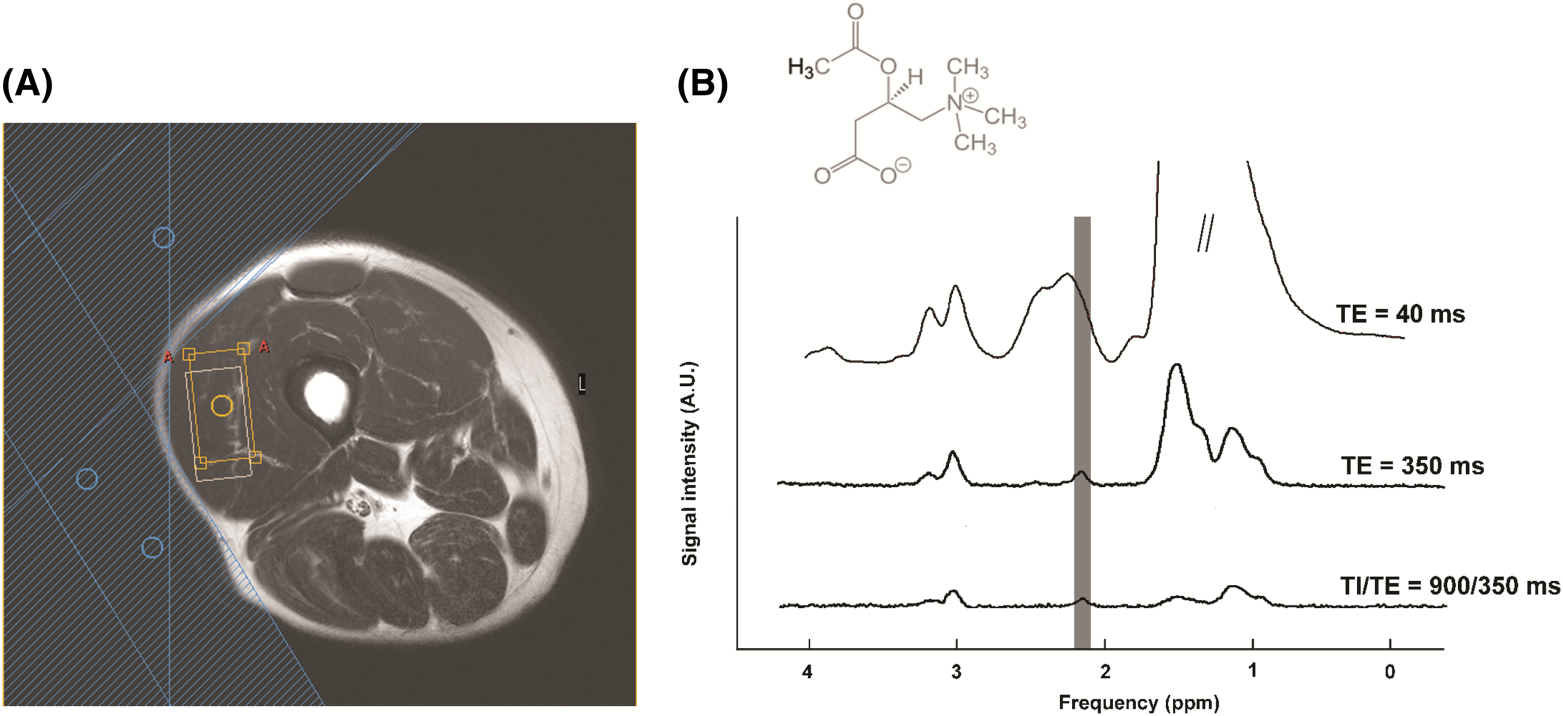
^1^H spectrum of the skeletal muscle acetylcarnitine. (A) Scout image depicting the position of the voxel in vastus lateralis (orange box), possible chemical shift displacement artefact between water and lipid resonances (white box), and three outer volume suppression slabs applied (blue shaded area). (B) Chemical structure of acetylcarnitine and spectra acquired with short T_E_ of 40 ms (upper trace), long T_E_ of 350 ms (middle trace), and a combination of long T_E_ and T_1_ inversion editing (T_E_ = 350 ms, T_I_ = 900 ms; lower trace). Improvement in detection and spectral resolution of acetyl carnitine methyl moiety at 2.13 ppm can be observed

Acetylcarnitine can be quantified from either the methyl peak of the acetyl moeity at 2.13 ppm (at any field strength) or the TMA group of the carnitine moiety at 3.17 ppm (ultrahigh‐field systems only) (Figure 9). These peaks resonate in a relatively crowded chemical shift range and are obscured by lipids at 2.2 ppm and carnitine/choline at 3.20 ppm, respectively, when acquired using conventional short T_E_ MRS. Although overlapped by lipid, the singlet methyl group signal at 2.13 ppm can be detected and quantified by exploiting the difference in their T_2_ relaxation times.^49^ The T_2_ of the methyl group of acetylcarnitine was estimated to be 265 ms at 3 T^49^ and 130 ms at 7 T,^50^ which is significantly longer than that of which is significantly longer_2_ lipid groups (2‐2.25 ppm) at these field strengths. The use of a long echo time (350 ms) sequence leads to a suppression of the overlapping lipid resonances and thus enhanced visibility of the acetylcarnitine singlet at 2.13 ppm. With such a long T_E_, acetylcarnitine can be detected in vastus lateralis at rest. Using this technique, a strong association between lower resting acetylcarnitine levels and whole body insulin resistance has been demonstrated. An equivalent long T_E_ approach has been applied at 7 T,^50^ and this study also proved applicability of the method in the soleus muscle. Since the T_1_ of acetylcarnitine is ~ 2000 ms (Table 1),^49^, ^50^ a relatively long T_R_ (≥6000 ms) should be used to avoid T_1_ saturation.

Even although a long T_E_ leads to relative decay of the overlapping lipid signal, difficulties may arise in subjects with high amounts of IMCL/EMCL, especially since these subjects are also characterized by relatively low acetylcarnitine levels. In these cases, a spin‐lattice relaxation‐ based editing approach can further improve the visibility of the acetylcarnitine signal.^100^ In subjects with very high amounts of IMCL/EMCL, the combination of a long T_E_ with the T_1_ editing method resulted in an uncontaminated acetylcarnitine peak (Figure 9).

With long T_E_ sequences, relatively large voxels (≥~ 20 mL) should be used to maintain a reasonable SNR. Care should be taken to place the voxel within a single muscle, to avoid (partial) inclusion of muscle groups with a different fiber composition and acetylcarnitine content. The use of a spin echo‐based localization sequence (ie, PRESS, semi‐LASER) is preferred due to the higher intrinsic SNR.

Similar to carnosine, since shimming from the manufacturer is often optimized for brain where no large fat signals are expected, phase‐sensitive shimming algorithms might fail in the fat‐containing large voxel for acetyl‐carnitine. A relatively small adaption of shimming voxel, alternative shimming or manual shim readjustment procedure might solve the problem.

Both water and methyl signal of tCr can be used as internal references to quantify absolute concentrations of acetylcarnitine. However, since the T_2_‐weighting of the water signal is very high and is very susceptible to small variations in water T_2_, the water reference signal should be obtained with a short T_E_ (≤30 ms) acquisition. When tCr is used as an internal reference, a correction factor for the effects of residual dipolar coupling on the splitting pattern of this resonance and its evolution due to prolonged T_E_ should be included. For exercise studies that could include changes in muscle hydration, tCr is preferred as a reference.

### 6.3 Physiological considerations for the study design

Resting levels of acetylcarnitine have been found to be higher in the vastus lateralis compared with soleus muscle in healthy, lean subjects.^50^ In agreement with the study of Lindeboom et al,^49^ acetylcarnitine concentrations have been associated with training status, increased insulin sensitivity and higher mitochondrial function. Diurnal changes of acetylcarnitine levels likely as a function of food intake and/or exercise have also been observed.^50^ Studies quantifying acetylcarnitine in biopsies show a very rapid increase with exercise^101^ and, furthermore, also report dependence on the macronutrient composition of the diet,^102^ with resting acetylcarnitine levels increasing with a high‐fat diet. A recent MRS‐based study shows that acetylcarnitine is increased after carnitine supplementation.^103^ Together, these findings emphasize that concentrations of acetylcarnitine respond dynamically to changes in metabolism and we therefore advocate a strict standardization of physical activity, timing of measurement and dietary conditions when resting levels of acetylcarnitine are measured. It is well documented that high‐intensity exercise strongly stimulates acetylcarnitine production,^101^ therefore, high‐intensity exercise protocols are recommended to maximize acetylcarnitine concentrations in dynamic protocols. Acetylcarnitine production was also reported to be higher when exercise was combined with intake of glucose prior to exercise although resting levels were decreased prior to exercise.^104^

Specific recommendations for ^1^H MRS of acetylcarnitine are listed in Table 5.

**TABLE 5:** Recommendations for ^1^H MRS of acetylcarnitine

Acquisition	Large voxel still must be placed in one muscle group to avoid inclusion of different fiber composition and acetylcarnitine content.
	Long T_E_‐protocols (e.g., 350 ms) allows detection of acetylcarnitine at rest (without the need of exercise) and irrespective of the field strength.
	Strong lipid signals can be reduced by the application of longitudinal relaxation time editing.
	Long T_E_ spectra require a relatively large voxel (min. 20 mL, preferably using PRESS or sLASER) and a sufficient number of acquisitions (64‐256) to achieve optimal SNR.
	Sufficiently long T_R_ (≥6000 ms) should be used to avoid T_1_ saturation.
Processing and /Quantitation	The singlet signal at 2.13 ppm is better suited for acetylcarnitine detection and quantification than the 3.17 ppm peak.
	While absolute concentration of acetylcarnitine can be based on either water or total creatine (tCr, 3.03 ppm), it is preferable to use tCr during exercise studies.
	Caveats for quantitation: small variations of water relaxation times have a strong effect and tCr must be corrected for residual dipolar effects.
Physiology and /Preconditioning	Studies of resting acetylcarnitine levels require a standardized preconditioning: 2 days omitting strenuous exercise and at least 1 day defined diet without large variations in macronutrient composition.
	MR examinations should be planned at the same time during the day to avoid effects of diurnal variations.
	Volunteers must rest for half an hour before the measurement.
	Intensity, duration, muscle investigated and fed/fasted state must be standardized and documented in exercise protocols.
	High‐intensity exercise protocols (in combination with glucose intake) are recommended to maximize acetylcarnitine concentrations.

## 7 ^1^H‐MRS OF DEOXYMYOGLOBINE AND LACTATE

In this final section, we combine the ^1^H MRS‐based assessment of lactate and deoxymyoglobin, each of which is produced in a physiological response of skeletal muscle, to exercise load and/or ischemic conditions. These two crucial metabolites both require MRS detection methods that go beyond simple straightforward single voxel localization and acquisition, limiting the widespread application outside of highly experienced MRS groups.

As already stated in the Introduction, the signal of deoxymyoglobine (dMb) appears in the downfield region of ^1^H MR spectra (78 ppm) of the skeletal muscle as a result of either increased oxygen demand or reduced oxygen supply. However, rather low concentrations (<0.5 mM) and very short T_2_ relaxation times (≤10 ms) make its detection dependent on surface‐coil localization and free‐induction‐decay (FID)‐based acquisition techniques.^24^, ^105^ On the other hand, short T_1_ (<10 ms) allow for short repetition times (TR of ~ 100 ms), and still reasonable T_2_ relaxation times at 1.5 T (of 10 ms) enabled FID‐based 1D and 2D CSI localization.^106^ A time resolution of several minutes was achieved but the in‐plane resolution remained rather crude and did not allow for exact localization of the specific muscle group. Nevertheless, high temporal resolution (≤ 5 seconds) of FID‐based topical localization experiments supports the characterization of dMb dynamics during exercise, ischemia and following recovery.^107^ Combined and interleaved experiments allowed for a direct comparison with the localization, onset and time course of changes in arterial spin labeling, ^31^P MRS postexercise dynamics,^108^ BOLD^109^ and NIRS^110^ signals. These sophisticated approaches reveal the potential interaction of dMb with tissue perfusion, intracellular pO_2_, O_2_ fluxes and tissue Mb concentration. In an extended experimental set‐up using two separated surface coils, differences in the amplitude of deoxygenation and time constants of reoxygenation were detected in distal vs. proximal locations of the calf in healthy volunteers as well as in patients with peripheral arterial disease.^24^ While all of these applications show a clinical potential of ^1^H‐MRS to monitor muscle perfusion, they also emphasize the importance of strict standardizations (locations of acquisition and physiological conditions) which need to be respected in future longitudinal and cross‐sectional studies.

Lactate is a product of an anaerobic glycolytic process and can also be detected by ^1^H‐MRS^19^, ^111^ under ischemic conditions. Ultrahigh fields (≥7 T) offer the resolution of CH‐group signal at 4.1 ppm^19^ in vivo, but the principal signal, a methyl group doublet at 1.35 ppm, is usually hidden underneath strong lipid resonances, which hinders accurate detection by simple PRESS‐ or STEAM‐based SVS methods. Spectral editing methods exploiting the ^1^H‐^1^H couplings within the molecule enable unambiguous detection and quantification of lactate.^20^ In this case, “single shot” double‐quantum filter methods^20^, ^112^ are preferred over single‐quantum editing approaches,^84^, ^111^ the latter being sensitive to muscle motion between the two editing excitations. In both cases, the choice of optimal sequence timing (T_E_) is crucial for a correct quantification and should consider the orientation dependence of residual dipolar coupling^20^, ^113^ and of biexponential behavior of spin‐spin relaxation,^113^, ^114^ which results from a compartmentation of lactate in intra‐ and extracellular spaces.

## 8 CONCLUSIONS

^1^H‐MRS of skeletal muscle can yield an abundance of information about the physiology, metabolic health and pathology of tissue. In this article, we have discussed how muscle‐specific morphology and physiology impact the resolution and appearance of muscle ^1^H‐MRS and have generated a series of recommendations to assist new users in performing such studies.

While an anatomically correct volume selection is important for all organs, it is mandatory for ^1^H‐MRS of skeletal muscle. The strong orientation dependence changes the spectral features of different muscles so much that a fit and an interpretation are invalid without an appropriate localization. The separation of EMCL and IMCL resonances, the residual dipolar coupling of several metabolites (including the methyl group of Cr/phosphocreatine that are often used as an internal concentration standard) change significantly with localization and change the spectral pattern beyond simple concentration differences.

By combining the recommendations in this article we have tried to emphasize these specifics of muscular ^1^H‐MRS. If these specific recommendations for skeletal muscle together with good general practice of ^1^H‐MRS are applied, abundant physiological and pathological information can be obtained.
